# Unraveling the Complexities of Idiopathic Multicentric Castleman Disease and Its Multi-systemic Associations: A Case Report

**DOI:** 10.7759/cureus.64935

**Published:** 2024-07-19

**Authors:** Aadi R Palvia, Prince Saha, Akshay Rahul Nandi, Abhiram Rao Damera, Aditya Suresh

**Affiliations:** 1 Internal Medicine, Kharghar Medicity Hospital, Navi Mumbai, IND; 2 Internal Medicine, California Institute of Behavioral Neurosciences & Psychology, Fairfield, USA; 3 Internal Medicine, Dr. D.Y. Patil Medical College, Hospital & Research Centre, Pune, IND; 4 Internal Medicine, Dr. B. R. Ambedkar Medical College, Bengaluru, IND; 5 Internal Medicine, Mediciti Institute of Medical Sciences, Hyderabad, IND; 6 General Medicine, Sri Ramachandra Institute of Higher Education and Research, Chennai, IND

**Keywords:** imcd, tocilizumab, siltuximab, antiphospholipid syndrome, human herpesvirus 6, idiopathic multicentric castleman's disease, castleman's disease

## Abstract

Castleman disease (CD) comprises a rare spectrum of disorders characterized by benign lymphoepithelial proliferation, classified into unicentric and multicentric forms. The idiopathic multicentric Castleman disease (iMCD) subtype, specifically, is challenging to diagnose and treat due to its variable manifestations and unpredictable disease course. We report a case of a 23-year-old female with a history of iron deficiency anemia presenting with concurrent antiphospholipid syndrome (APS) and human herpesvirus-6 (HHV-6) positivity. Investigations revealed a gastric mass, with a biopsy suggestive of the plasma cell variant of CD. This case report aims to understand the possible association of HHV-6 positivity with CD and the significance of diagnosing APS early in patients with the disease. Treatment with siltuximab and tocilizumab proved effective, highlighting the role of interleukin 6 (IL-6) in the elusive etiology of this condition.

## Introduction

Castleman disease (CD) encompasses a spectrum of rare lymphoproliferative disorders characterized by benign lymph node enlargement that is further clinically classified into two categories based on regional involvement: unicentric Castleman disease (UCD) and multicentric Castleman disease (MCD) [1]. MCD manifests primarily in two ways: idiopathic multicentric Castleman disease (iMCD) and MCD linked with human herpesvirus-8 (HHV-8). The iMCD subtype presents unique diagnostic and therapeutic challenges due to its heterogeneous clinical manifestations and variable disease course. Although the exact cause of iMCD is unknown, it has been associated with autoimmune diseases, possible viral triggers such as HHV-8, and genetic abnormalities that promote the overproduction of cytokines, especially interleukin-6 (IL-6). The symptoms of iMCD include hyperplastic germinal centers, dysregulated lymphocyte proliferation, and enlarged lymph nodes. From a histopathological viewpoint, the changes are reactive and caused by an excessive level of cytokines rather than tumor development. Middle-aged individuals, particularly men, are the main target population for iMCD, which has a diverse clinical severity that can range from minor lymphadenopathy to systemic inflammation and organ involvement. Excluding infections, autoimmune diseases, and cancers that resemble the histological characteristics of iMCD is necessary for the diagnosis. Accurate illness management is aided by routine monitoring of organ function and cytokines. To reduce inflammation and symptoms, treatment comprises IL-6 blocking (e.g., tocilizumab, siltuximab). Although patient responses differ, supportive treatment targets organ problems with the goal of symptom alleviation and possible remission [2]. Given the rarity of our case, documenting and disseminating our clinical experience is imperative to enhance understanding and management strategies for similar cases in the future.

## Case presentation

A 23-year-old female presented to the emergency department with complaints of dizziness, lightheadedness, shortness of breath, and generalized muscle pain. She was a known case of severe iron deficiency anemia (IDA) and was being treated with blood transfusions. Despite multiple transfusions, her symptoms worsened, prompting her to seek medical attention. She reported to the hospital that she was feeling fatigued, and her hemoglobin was found to be 4.9 g/dL. 

On examination, the patient was alert and oriented to person, place, and time, appearing well without acute distress. Examination of the head, ears, nose, and throat revealed normal findings. There were no sinus complaints or oropharyngeal issues noted. However, pallor was present in the eyes. Cardiovascular and pulmonary examinations are unremarkable, with normal S1/S2 sounds, regular rate and rhythm, and no murmurs, wheezes, rales, or rhonchi on lung auscultation bilaterally. The abdomen was soft, without organomegaly, tenderness, or ascites. Neurologically, the patient exhibited dizziness but no gross deficits or asterixis. Skin examination showed no rash or lesions, and there was no edema in the extremities.

Laboratory investigations are shown in Table 1. It revealed severe anemia, with a hemoglobin level of 4.9 g/dL, and other hematological abnormalities. Erythrocyte sedimentation rate (ESR) and C-reactive protein (CRP) levels were elevated. Liver and kidney function tests were unremarkable.

**Table 1:** Routine blood investigations on admission Bold values indicate abnormal values.

Test	Result	Unit	Normal Range
Hemoglobin	4.9	g/dL	12-16
Red blood cell count	2.22	million/cumm	4.0-5.2
Hematocrit	17.6	%	36-44
Mean corpuscular volume (MCV)	79.8	fL	77-95
Red cell distribution width	55.1	%	11.5-14.5
White blood cell count	8	10^3^/uL	4.5-11
Erythrocyte sedimentation rate	58	mm	0-20
C-reactive protein	62	mg/L	0-6
Total bilirubin	0.9	mg/dL	0.2-1.2
Direct bilirubin	0.4	mg/dL	0.10-0.40
Indirect bilirubin	0.5	mg/dL	0.10-1.00
​​Aspartate transaminase	38	IU/L	5-40
Alanine transaminase	32	IU/L	5-40
Alkaline phosphatase	96	IU/L	40-125
Serum creatinine	0.7	mg/dL	0.5-1.5
Blood urea nitrogen	10	mg/dL	8-21

**Figure 1:**
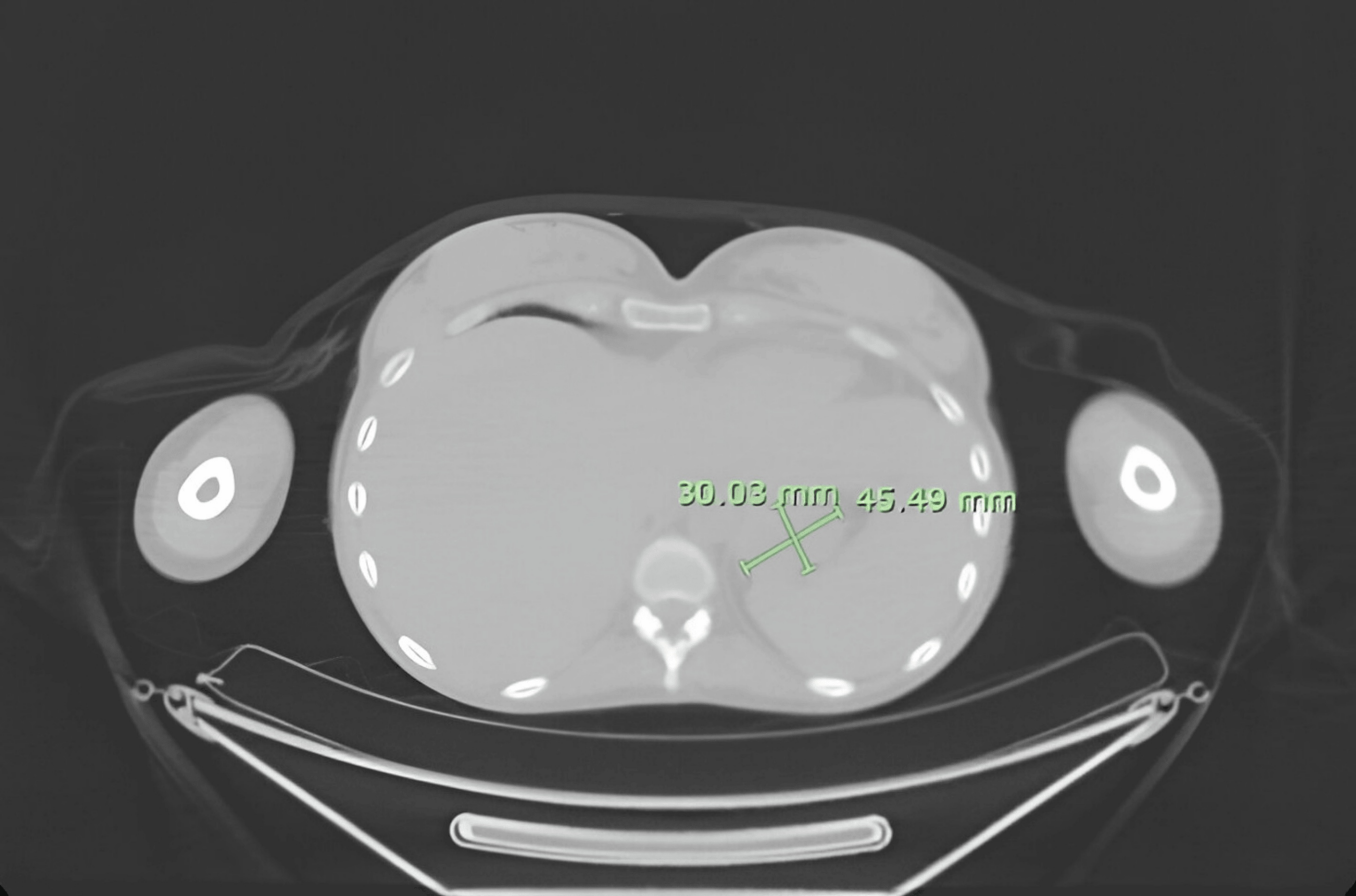
Computed tomography scan showing a gastric mass measuring 30.03 x 45.49 mm

**Figure 2:**
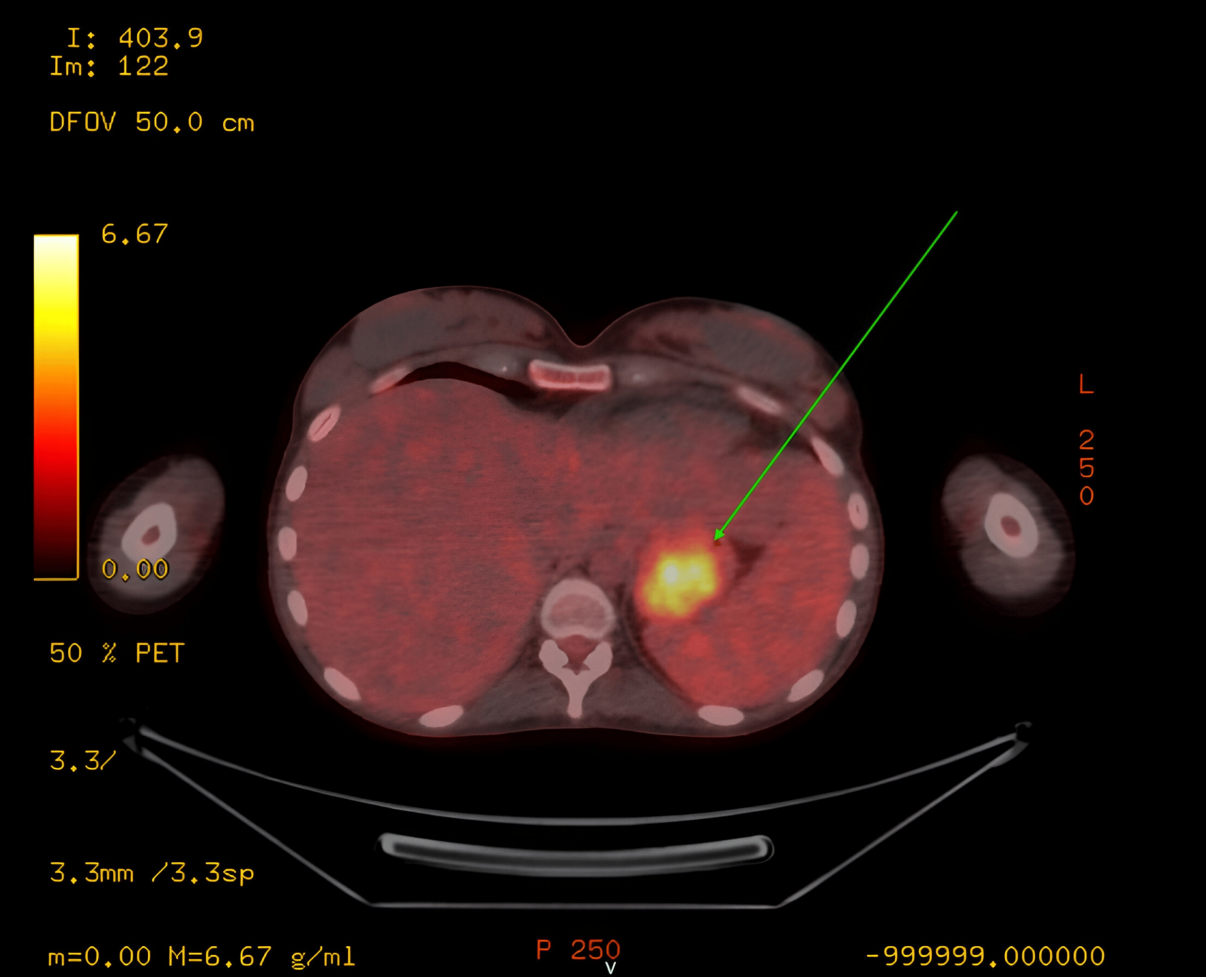
Positron emission tomography scan The arrow shows a gastric mass.

Computed tomography (CT) scan shown in Figure 1 and positron emission tomography (PET) scan shown in Figure 2 revealed a gastric mass. The size of the gastric mass in the CT scan was 30.03 x 45.49 mm.

**Figure 3:**
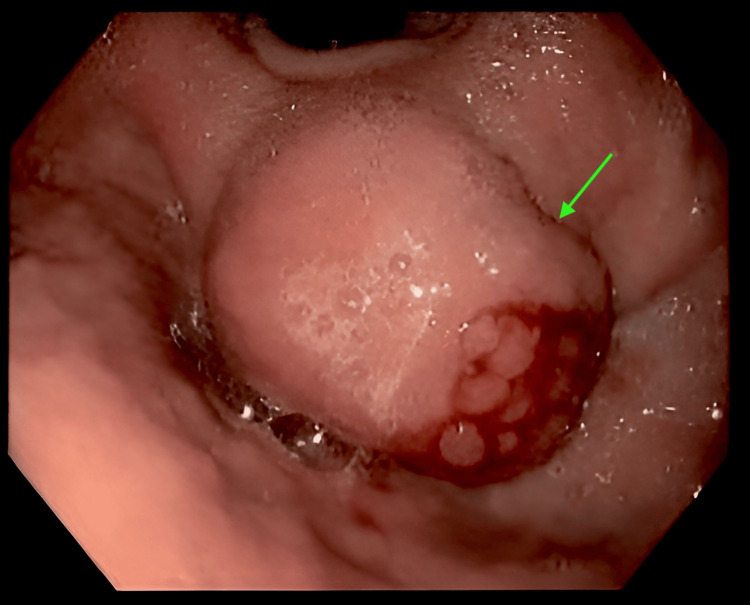
Upper gastrointestinal endoscopic image of the gastric fundus revealing a mass

The patient was comprehensively investigated for other causes of anemia as it did not respond to the standard therapy for IDA, which included an upper gastrointestinal endoscopy. During the procedure, a gastric mass (Figure 3) was found in the fundus, which on biopsy revealed patchy lymphoid infiltrates and fibrosis, suggestive of CD. The patient also suffered from a splenic infarction secondary to antiphospholipid syndrome (APS).

A gastric biopsy showed patchy lymphoid infiltrate and immunostained positive for B and T cells (CD3, CD20, BCL-2, CD-138). Kappa and Lambda in situ hybridization showed sheets of polyclonal plasma cells. Epstein-Barr encoding region in situ hybridization and HHV-8 were negative. Further testing revealed positive HHV-6 IgG. The patient was started on siltuximab and tocilizumab for suspected CD. Due to her coagulation disorder, warfarin was replaced with heparin. After the initiation of the treatment, ESR and CRP levels started to normalize. The patient's serum IL-6 levels returned to normal, and her condition significantly improved. After continuing treatment, follow-up revealed preserved normal IL-6 levels and reduced symptoms. She is currently under close monitoring and follow-up to assess her long-term response to treatment and manage her complex medical condition.

## Discussion

CD is an infrequent condition characterized by benign enlargement of lymph nodes, detected through angiofollicular lymph node hyperplasia upon histological examination. Initially documented by Castleman and colleagues in 1956, it presented as confined benign lymphadenopathy [1]. It is essential to contemplate CD in the differential diagnosis of localized or diffuse lymphadenopathy, whether or not accompanied by systemic symptoms. This case study strives to provide new perspectives on this uncommon and relatively harmless condition. Despite resembling lymphoma clinically, CD differs significantly in terms of histology, prognosis, and available treatment options.

From a clinical perspective, the condition manifests in two ways: (I) localized, originally recognized by Castleman, and more prevalent; (II) multicentric, impacting various locations, initially reported by Gaba et al. in 1972 [3]. The multicentric type is subdivided into three: (I) POEMS-associated MCD with specific symptoms; (II) HHV-8-associated MCD; and (III) idiopathic MCD (iMCD), the most common type. iMCD is classified into three categories: (I) iMCD is associated with TAFRO, which presents with thrombocytopenia, anasarca, fever, renal dysfunction, and organomegaly; (II) iMCD with idiopathic plasmacytic lymphadenopathy, mainly characterized by thrombocytosis; (III) iMCD, not otherwise specified [4].

From a histological standpoint, the disease presents three primary types: (I) The hyaline vascular type is defined by the formation of lymphoid follicles that create a layered or "onion skin" pattern around a hyalinized vessel located in the core of the follicle. This type is more common in the localized manifestation of the disease. (II) The plasma cell variation is characterized by mature plasma cells forming sheets in interfollicular tissues around bigger germinal centers with noticeably reduced vascularity. This variation is predominantly linked to the multicentric form of the disease. (III) Another histological variant displays a combination of features and is also seen in MCD [5].

The etiology of CD is still unknown, despite several proposed causes in the literature. Increasing research indicates a possible connection to viral infections. Multiple studies show increased IL-6 production in CD lesions [6,7]. High IL-6 levels stimulate the liver to produce more hepcidin, which impairs iron absorption and causes IDA [8].

This case report also reports the presence of APS with splenic infarcts in the patient. APS is a systemic autoimmune disorder known for causing blood clots, pregnancy complications, and the presence of antiphospholipid antibodies [9]. Individuals with APS commonly exhibit deep venous thrombosis, livedo reticularis, or stroke. Additionally, they may experience thrombocytopenia, myocardial infarction, and, in rare cases, catastrophic APS, which can be life-threatening [10,11]. APS could be a significant yet unrecognized main cause of TAFRO syndrome, appearing as an iMCD. TAFRO syndrome is a diverse condition that encompasses iMCD-TAFRO, cancer, infection, and autoimmune disease [12]. It is crucial to thoroughly identify the main cause of TAFRO syndrome for appropriate therapy and monitoring strategies. Further research is needed to verify if APS is the root cause of TAFRO.

CD often presents with B symptoms, including fever, weight loss, night sweats, and exhaustion, along with physical signs such as splenomegaly or hepatomegaly and fluid retention (edema, anasarca, and pleural effusion). Lymph node histopathologic screening distinguishes between the involvement of a single node (indicative of UCD) or many nodes (suggestive of MCD), while also excluding other possible illnesses. Significant characteristics of the lymph nodes are recorded. Laboratory markers such as elevated CRP or ESR, anemia, thrombocytopenia or thrombocytosis, hypoalbuminemia, and renal failure or proteinuria assist in the diagnosis process. Furthermore, serology tests are performed for HIV and HHV-8, and HHV-8 DNA in peripheral blood is examined using polymerase chain reaction (PCR) to detect connections with CD. This case involves HHV-6 DNA, highlighting the current study to understand the complete range of the disease. According to a study conducted by David C. Fajgenbaum et al., HHV-6 signals proinflammatory cytokines, and CD may develop as a result of HHV-6's modification of immunological activities [1].

Treatment options for MCD include surgery, cytotoxic chemotherapy with or without corticosteroids, and autologous stem cell transplantation, each resulting in different outcomes. Improved results are seen by focusing on the CD20 and IL-6 pathways, as well as suppressing HHV-8 replication. Monoclonal antibodies such as tocilizumab and siltuximab, which interfere with the IL-6 signaling pathway, are highly effective and recommended as the initial treatment for symptomatic patients who do not test positive for HIV or HHV-8. Treatment with chemotherapy and immunomodulatory medicines is only used in cases of relapse. It is important to emphasize that not all cases of CD respond to treatment, and a cure is not always guaranteed [13].

## Conclusions

This case report of a 23-year-old female patient who has both iMCD and APS highlights the complex nature of the relationship between autoimmune disease and lymphoproliferative disorders. The rare nature of this case, which is suspected to have been brought on by HHV-6, increases the difficulty in diagnosing this illness. The combination of severe anemia, a stomach mass, and CD coexisting with APS was discovered throughout the patient's clinical course. This case demonstrates the value of multidisciplinary management in handling such rare presentations. The presence of a viral infection and elevated levels of IL-6 demystify the potential etiologies of CD. Tocilizumab and siltuximab are used to treat this condition, which shows the importance of therapeutic interventions targeting the IL-6 pathway. After receiving siltuximab and tocilizumab, the patient's symptoms significantly improved, and normalized ESR, CRP, and IL-6 levels were maintained at follow-up. By documenting this case, our aim is to advance understanding of coexisting autoimmune and lymphoproliferative diseases. To enhance outcomes in instances similar to this clinical experience, additional investigation and collaborative endeavors are required to formulate diagnostic methodologies and therapeutic approaches.
